# Changes in Respiratory Syncytial Virus‐Associated Hospitalisations Epidemiology After Nirsevimab Introduction in Lyon, France

**DOI:** 10.1111/irv.70054

**Published:** 2024-12-19

**Authors:** Cécile Chauvel, Côme Horvat, Etienne Javouhey, Yves Gillet, Juliette Hassenboehler, Claire Nour Abou Chakra, Corinne Ragouilliaux, Franck Plaisant, Dominique Ploin, Marine Butin, Jean‐Sebastien Casalegno, Marta C. Nunes

**Affiliations:** ^1^ Center of Excellence in Respiratory Pathogens (CERP) Hospices Civils de Lyon (HCL) Lyon France; ^2^ Centre International de Recherche en Infectiologie (CIRI), Équipe Santé Publique, Épidémiologie et Écologie Évolutive des Maladies Infectieuses (PHE3ID), Inserm U1111, CNRS UMR5308, ENS de Lyon Université Claude Bernard Lyon 1 Lyon France; ^3^ Hospices Civils de Lyon (HCL) Hôpital Femme Mère Enfant, Services d'Urgences et de Réanimation Pédiatriques Bron France; ^4^ EA 7426 “Pathophysiology of Injury‐Induced Immunosuppression” University Claude Bernard Lyon 1 Lyon France; ^5^ Centre International de Recherche en Infectiologie (CIRI), Laboratoire StaPath, Inserm U1111, CNRS UMR5308, ENS de Lyon Université Claude Bernard Lyon 1 Lyon France; ^6^ Hospices Civils de Lyon (HCL) Hôpital de la Croix Rousse, Maternité Lyon France; ^7^ Hospices Civils de Lyon (HCL), Centre Hospitalier Lyon Sud, Maternité Pierre‐Bénite France; ^8^ Centre International de Recherche en Infectiologie (CIRI), Laboratoire Vir'Path, Inserm U1111, CNRS UMR5308, ENS de Lyon Université Claude Bernard Lyon 1 Lyon France; ^9^ Hospices Civils de Lyon (HCL) Hôpital Femme Mère Enfant, Maternité Lyon France; ^10^ Hospices Civils de Lyon (HCL), Hôpital de la Croix‐Rousse, Centre de Biologie Nord Institut des Agents Infectieux, Laboratoire de Virologie Lyon France; ^11^ Vaccines & Infectious Diseases Analytics (VIDA) Research Unit, Faculty of Health Sciences University of the Witwatersrand Johannesburg South Africa

**Keywords:** Beyfortus, effectiveness, LRTI, monoclonal antibody, Nirsevimab, paediatrics, real‐world evidence, RSV

## Abstract

**Background:**

Respiratory Syncytial Virus (RSV) is a major health concern, particularly for infants. In France, Nirsevimab, a long‐acting monoclonal antibody to prevent RSV‐associated lower respiratory tract infections (LRTI) was available from September 2023. We described RSV‐associated LRTI hospitalisations during the 2023–2024 season among infants younger than six months born at the Hospices Civils de Lyon (HCL), and evaluated the effectiveness of Nirsevimab against RSV‐LRTI hospitalisation.

**Methods:**

This observational study included infants born and hospitalised at the HCL during the 2023–2024 season, along with pre‐COVID‐19 and 2022–2023 seasons. Information on Nirsevimab immunisation status, clinical and perinatal variables were collected through routine care. Infants' characteristics and incidence rate of hospitalisation per 100 births during 2023–2024 were compared with the historical periods overall and by delay between birth and the onset of the RSV season. Nirsevimab effectiveness was computed by the screening method.

**Results:**

During the 2023–2024 season, 83 infants younger than six months were hospitalised with an RSV‐associated LRTI. Compared with the historical periods (640 pre‐COVID‐19 and 123 in 2022–2023), these infants were older. Incidence rate for infants born during the period when immunisation was available were lower than the previous seasons; incidence rate ratios were 0.45 (95% confidence interval [CI]: 0.33; 0.62) in 2023–2024 compared with pre‐COVID‐19 period and 0.53 (95%CI: 0.36; 0.77) compared with 2022–2023 season. Nirsevimab effectiveness was 78.3% (95%CI: 55.9; 89.5) with a coverage of 79.3% in the two main HCL maternities.

**Conclusions:**

High Nirsevimab coverage and effectiveness were estimated in a real‐world setting. A change in the age distribution of RSV‐associated LRTI hospitalisations in 2023–2024 was noted compared with historical seasons.

## 1 Introduction

Respiratory Syncytial Virus (RSV) is a major health concern, particularly for infants and young children. Epidemiological studies reveal that about one‐third of infants contract symptomatic RSV infections in their first year of life [1, 2]. Although many infections are mild and self‐limiting, RSV is also a leading cause of acute lower respiratory tract infections (LRTI) among young children [3]. In Europe, it has been estimated that every year an average of 10 children younger than five years per 1000 are hospitalised due to RSV disease [2]. The risk of hospitalisation due to RSV peaks in the first few months of life, decreasing as children age.

Nirsevimab (Beyfortus, AstraZeneca and Sanofi), a long‐acting monoclonal antibody, has been developed to prevent RSV‐associated LRTI. It received marketing authorization from the European Medicines Agency in October 2022, for use in newborns and children during their first RSV season [4]. Following this, on 15 September 2023, France launched a national immunisation campaign targeting newborns in maternity wards before discharge, becoming one of the first countries to implement such a measure [5].

The RSV seasons in Northern Hemisphere countries, including France, typically last from November to mid‐February, peaking around the end of December [6]. Between 2010 and 2018, France averaged 50,878 hospitalisations coded as RSV‐associated annually among children [7]. Although the incidence of RSV dropped significantly during the initial year of the COVID‐19 pandemic, rates by the end of the 2021–2022 season returned to pre‐pandemic levels [8].

This study aimed to evaluate the effectiveness of Nirsevimab in preventing RSV‐associated LRTI hospitalisations within the first six months of life in a real‐world setting and to describe the incidence rate of RSV‐associated LRTI hospitalisations during the 2023–2024 season among infants born at the Hospices Civils de Lyon (HCL) maternities, making comparisons with historical data.

## 2 Methods

### 2.1 Study Population

We conducted a retrospective observational study among infants admitted to the HCL with an RSV‐associated LRTI and who were born at one of the three HCL maternities. The HCL is the only public tertiary hospital serving the city of Lyon (France) and surrounding urban area, corresponding to a population of 1.4 million inhabitants in 2020 [9]. Infants younger than six months, born at the HCL and hospitalised during the 2023–2024 RSV season with a positive RSV‐reverse transcription polymerase chain reaction (RT‐PCR) were automatically identified using the hospital's birth and virology laboratory databases, constituting the main study population. Secondary study populations included infants with the same characteristics (born at the HCL and hospitalised at the HCL with a positive RSV RT‐PCR result during the first six months of live) hospitalised in the previous seasons of 2015–2016 to 2022–2023, excluding the two COVID‐19 pandemic seasons, (i.e. 2020–2021 and 2021–2022 due to changes in RSV epidemiology during the pandemic [10]). The selection methodology has been previously reported [11, 12].

The Nirsevimab immunisation campaign at the HCL began on 15 September 2023, as across France, and infants were offered immunisation at the maternity before being discharged after birth. Each of the three hospital maternities kept individual logs of the Nirsevimab doses administered. Weekly immunisation coverage in two maternities, and overall coverage in the third maternity from 15 September to 31 December 2023, were available. Neonates transferred to the neonatal intensive care unit directly after birth were not included in the Nirsevimab coverage calculation since some of them were not discharged from hospital during the epidemic season.

All electronic medical files of the RSV‐associated hospitalisations were manually reviewed to collect clinical observations. Information on vital measurements, clinical observations, therapeutics and paediatric intensive care unit admission were collected. Respiratory support, whether non‐invasive or invasive, was always performed at the intensive care unit. Feeding support was either enteral nutrition or parenteral fluid administration. For infants hospitalised during the 2023–2024 season, Nirsevimab administration information, including date of immunisation, was collected by i) inspecting all the electronic birth records, where this information was recorded if immunisation occurred before being discharged, and ii) reviewing the electronic medical files of the RSV‐associated hospitalisations when parents were actively asked about the infants' immunisation status. Infants were considered immunised if they received Nirsevimab more than seven days before hospital admission.

Infants without respiratory symptoms, with sudden infant death syndrome, with nosocomial infection (for whom the respiratory symptoms started after two days of hospitalisation), hospitalised less than 24 h before transferred to another hospital (in case of bed saturation for the less severe infants and those at a lower risk), and infants whom parents refused the retrospective survey (see Ethics statement) were excluded.

### 2.2 Statistical Analyses

Categorical variables were described by their counts and frequencies and compared using Pearson's Chi‐squared test. Continuous variables were described by their median and inter‐quartile range (IQR) and compared by Wilcoxon rank‐sum test. All analyses were performed with the R statistical software, version 4.3.0 [13].

RSV seasons in our setting ranged from October to March. Infants hospitalised during the 2023–2024 season were compared with infants hospitalised during two historical periods, the (i) pre‐COVID‐19 seasons (2015–2020) and (ii) the 2022–2023 season. Incidence rate of RSV‐associated hospitalisations per 100 births for each of the three study periods and incidence rate ratios (IRR) comparing the 2023–2024 season with the two historical periods were calculated using the median unbiased estimation and exact 95% confidence intervals (CI) [14]. The start of each RSV season in the Auvergne‐Rhône‐Alpes region was identified using the data available on the Santé Publique France website [6]. Incidence rate and IRR were stratified by delay in months between birth and the corresponding RSV season onset for each study period. For the 2023–2024 season, infants born more than two months before season onset were born before the start of the Nirsevimab immunisation campaign, and thus were not eligible for immunisation. Since the first day of the Nirsevimab immunisation campaign was a Friday, infants born during this week (week 37, 2023) were removed from the hospitalisation incidence analysis, as well as those born in the corresponding weeks in relation to season onset for all other years.

Infants hospitalised with an RSV‐associated LRTI during the 2023–2024 season and born after 15 September 2023 were stratified in immunised (Nirsevimab administered) and non‐immunised (no Nirsevimab) groups. Disease characteristics (age at admission, gender, being born preterm, need for supplemental oxygen therapy or feeding support and intensive care unit admission) were compared between these two groups.

The effectiveness of Nirsevimab against RSV‐associated LRTI hospitalisations among infants born at the HCL between 15 September and 31 December 2023, was calculated using the Farrington's screening method based on (i) the number of RSV‐associated hospitalisations, (ii) the number of infants immunised among these hospitalisations, and (iii) the percent of newborns in the birth cohort who were immunised [15]. During September to December 2023, one of the three maternities had half the number of births of the other two, and did not record weekly Nirsevimab administration numbers. Thus, this maternity was not included in the main effectiveness analysis. Nirsevimab effectiveness was calculated as 1 minus the odds of immunisation among the RSV infants compared with the immunisation coverage in the birth cohort, with adjustment for week of birth, as Nirsevimab coverage varied by week. Three sensitivity analyses were conducted: (i) using data from the two main maternities with adjustment on week of birth, excluding the first two weeks of Nirsevimab availability where coverage was below 70%, (ii) using data from the two main maternities adjusting for maternity only and (iii) using data from the three maternities adjusting for maternity. Neonates transferred directly after birth to neonatal intensive care unit were not included in the effectiveness analyses.

The STROBE guidelines were followed to report the study [16].

## 3 Results

**FIGURE 1:**
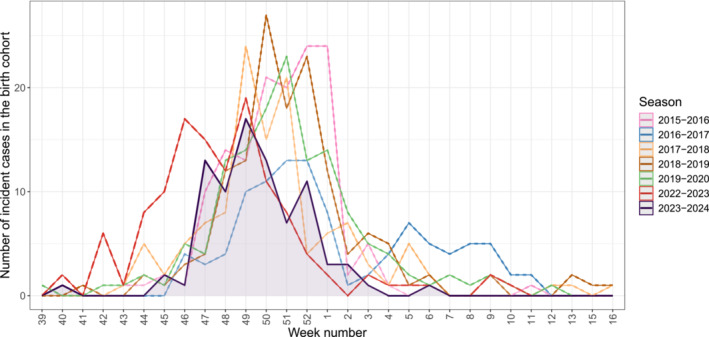
Epidemic curves of RSV‐associated lower respiratory tract infection hospitalisations among infants younger than six months born at the Hospices Civils de Lyon. Shaded area represents the RSV‐associated hospitalisations during the 2023–2024 season.RSV‐associated lower respiratory tract infection hospitalisations detected in the historical season: 2015–2016:141, 2016–2017:103, 2017–2018:119, 2018–2019:140, 2019–2020:137, 2022–2023:123.

Compared with the historical pre‐COVID‐19 seasons and the 2022–2023 season, the 2023–2024 RSV season at the HCL recorded a lower total number of RSV‐associated hospitalisations among infants younger than six months from the hospital birth cohort (Table 1). The 2023–2024 season was narrower and with a shorter peak compared with the previous seasons (Figure 1). The first RSV case was detected in the first week of October 2023, and the last detection was on the second week of February 2024. During the 2023–2024 RSV season, 83 infants younger than six months, born at one of the HCL maternities, were hospitalised with an RSV‐associated LRTI. Their median age at admission was 76 days (IQR: 40, 110), they were significantly older than the infants admitted during the pre‐COVID‐19 seasons (median age of 53 days, *p*‐value < 0.001) and the 2022–2023 season (median age of 51 days, *p*‐value = 0.003) (Table 1 and Supplementary Figure S1). During the 2023–2024 season, 46% of the hospitalised infants were males and 14% were born preterm, this was not statistically different to the findings from historical seasons (Table 1).

**TABLE 1:** Characteristics of infants younger than six months hospitalised with RSV‐associated lower respiratory tract infections and born at the Hospices Civils de Lyon.

	Pre‐COVID‐19 RSV seasons^a^		2022–2023 RSV season		2023–2024 RSV season
	*N* = 640	*p*	*N* = 123	*p*	*N* = 83
Median age at admission in days (IQR)	53 (27, 84)	< 0.001	51 (29, 88)	0.003	76 (40, 110)
Age group, *n* (%)		0.002		0.019	
0–30 days	181(28)		31 (25)		15 (18)
31–60 days	177 (28)		42 (34)		19 (23)
61–90 days	147 (23)		22 (18)		14 (17)
91–120 days	58 (9.0)		14 (11)		18 (22)
121–150 days	42 (6.6)		4 (3.3)		11 (13)
151–183 days	35 (5.5)		10 (8.1)		6 (7.2)
Male, *n* (%)	353 (55)	0.11	72 (59)	0.072	38 (46)
Pre‐term (< 37 weeks), *n* (%)	112 (18)	0.5	14 (11)	0.5	12 (14)
Very pre‐term (< 32 weeks), *n* (%)	24 (3.8)	> 0.9	5 (4.1)	> 0.9	3 (3.6)

*Note: p*‐Values compare the 2023–2024 season with the other two periods.

Abbreviation: IQR, interquartile range.

^a^ N per pre‐COVID‐19 season: 141 (2015–2016), 103 (2016–2017), 119 (2017–2018), 140 (2018–2019), 137 (2019–2020).

**FIGURE 2:**
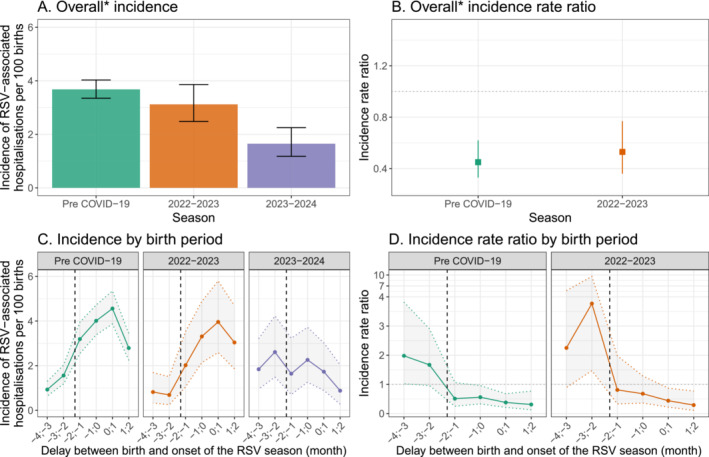
Incidence of RSV‐associated lower respiratory tract infection hospitalisations during the first six months of life among the Hospices Civils de Lyon birth cohort for pre‐COVID‐19, 2022–2023 and 2023–2024 seasons. *For 2023–2024 season, overall includes all infants born from 18 September to 31 December 2023 (−2 to 2 months). In panels B and D, the dotted vertical line corresponds to the first administration of Nirsevimab.

A total of 40 (48.2%) infants hospitalised with an RSV‐associated LRTI in 2023–2024 were born between 18 September and 31 December 2023. Correspondingly, the incidence rate of RSV‐associated LRTI for the infants born during this period was 1.65 per 100 births (95%CI: 1.18, 2.25), which was approximately half of the incidence during the pre‐COVID‐19 seasons (IRR: 0.45 [95%CI: 0.33, 0.62]) and the 2023–2024 season (IRR: 0.53 [95%CI: 0.36, 0.77]), (Figure 2A, Figure 2B and Supplementary Table S1). When stratifying by month of birth, significant reductions in 2023–2024 incidence rate compared with the historical seasons were noted for infants born from one month prior to one month after the RSV season onset (Figure 2C, Figure 2D and Supplementary Table S1). Conversely, infants born before the start of the Nirsevimab campaign in 2023 (i.e. three and four months before the RSV season onset) had higher incidences of hospitalisation compared with the historical seasons (Figure 2D and Supplementary Table S1). During the 2023–2024 season, two infants hospitalised with an RSV‐associated LRTI were born after 1 January 2024 (i.e. more than 2 months after the onset of the epidemic) and due to the low number were excluded from the incidence analysis.

Table 2 shows the demographics and clinical characteristics of the immunised and non‐immunised infants hospitalised with an RSV‐associated LRTI during the 2023–2024 season. Among the infants born at the HCL while Nirsevimab was being offered and who were hospitalised for an RSV‐associated LRTI during their first six months of life, 51.2% (21/41) had been immunised. The immunisation status was missing for one infant. All immunisations had occurred between 23 days and 89 days before hospital admission. Only age at hospitalisation was found to be significantly different, with older (median age of 46 days compared with 28 days) infants in the immunised group.

**TABLE 2:** Characteristics of infants hospitalised with an RSV‐associated lower respiratory tract infection born during Nirsevimab availability according to immunisation status.

	Immunisation	No immunisation	*p*
	*N* = 21	*N* = 20	
Median age at admission in days (IQR)	46 (39, 68)	28 (17, 44)	0.003
Preterm (< 37 gestational weeks), *n* (%)	3 (14)	1 (5)	0.6
Male, *n* (%)	8 (38)	9 (45)	0.7
Supplemental oxygen therapy, *n* (%)	10 (48)	7 (50)	0.4
Paediatric intensive care unit admission^a^, *n* (%)	5 (24)	7 (35)	0.4
Feeding support^b^, *n* (%)	16 (76)	16 (80)	> 0.9

Abbreviation: IQR, interquartile range.

^a^ In our hospital paediatric intensive care unit admission is performed when respiratory support is required, whether invasive or non‐invasive.

^b^ Feeding support is either enteral nutrition or parenteral fluid administration.

Between 15 September and 31 December 2023 (week 38 to week 52), the coverage of Nirsevimab immunisation was 80.1% at the three HCL maternities, and 79.3% among the two main maternities (Table 3). Including only the births at the two main maternities, Nirsevimab effectiveness against RSV‐associated LRTI hospitalisation was 78.3% (95%CI: 55.9, 89.5). During the first two weeks of Nirsevimab availability, immunisation coverage was below 70% in the two main maternities, increasing thereafter to 81.5%. A sensitivity analysis excluding these two weeks produced an effectiveness point‐estimate of 84.1% (95%CI: 66.6, 92.7) (Table 3).

**TABLE 3:** Nirsevimab effectiveness against RSV‐associated lower respiratory tract infection hospitalisations among infants younger than six months born at the Hospices Civils de Lyon.

	Weeks	Maternities	Effectiveness (%) [95% CI]	Global coverage (%)
Main analysis	38–52	2 main maternities	78.3 [55.9;89.5]^a^	79.3
Sensitivity analyses	38–52	2 main maternities	78.2 [55.6;89.4]^b^	79.3
	40–52	2 main maternities	84.1 [66.6;92.7]^a^	81.5
	38–52	All 3 maternities	75.6 [52.6;87.4]^b^	80.1

^a^ Adjustment on the birth week.

^b^ Adjustment on the maternity.

## 4 Discussion

During the initial season of 2023–2024 of Nirsevimab implementation in France, a high coverage of 80.1% was achieved among the HCL birth cohort. A difference in RSV hospitalisations compared with historical seasons was detected, notably an overall lower number of RSV‐associated hospitalisations were recorded, with an increase in older infants. A temporal association between Nirsevimab introduction and a decrease in the incidence rate of hospitalisation by at least 40% was specially recorded for infants born from one month prior to the RSV season onset, coinciding with one month after Nirsevimab started to be offered at birth in the maternity wards. A higher incidence was, however, detected among infants born before Nirsevimab availability, infants who were 3–4 months old at season onset, compared with the historical seasons. We are tempted to speculate that the prevention of a notable number of hospitalisations for RSV among very young infants during the 2023–2024 RSV season contributed to paediatric hospital resources not being overwhelmed, and as such older infants who would be discharged from the emergency room in prior seasons were admitted because bed availability [17]. Although not directly assessed by the study, this increase certainly indirectly contributed to improve the general care provided in the paediatric hospital. Future seasons will allow to confirm the reproducibility of our result. If confirmed, and whatever mechanisms explain this change (e.g., bed availability through hospitalisation reduction, changes in epidemics transmission dynamic), these patients are to be accounted for both preventive measures implementation and a larger part of the Nirsevimab‐era RSV hospital burden from now on. Extending the immunisation to infants born before 15 September 2023 could have prevented additional RSV‐related hospitalisations in these older infants. Notably, in clinical trials, Nirsevimab remained efficacious against RSV‐associated LRTI in infants through 150 days after immunisation [18]. A study from Luxembourg also described a decrease of 69% in the number of RSV‐associated hospitalisations among infants younger than six months after Nirsevimab implementation, compared with the 2022–2023 RSV season 2022–2023 RSV season [19].

Among the HCL birth cohort, Nirsevimab reduced the risk of RSV‐associated LRTI hospitalisations by 78.3%. This estimate is similar to the Nirsevimab efficacy against RSV‐associated LRTI hospitalisations reported in a Phase 3 clinical trial in healthy late‐preterm and term infants of 76.8%, and a pragmatic randomised controlled trial, which estimated an efficacy of 83.2% [20, 21]. Post‐implementation studies from Spain also reported that Nirsevimab substantially reduced infant RSV‐associated hospitalisations including more severe episodes [19, 22]. In France, Nirsevimab effectiveness was estimated at 75.9% for RSV bronchiolitis hospitalised in paediatric intensive care units [23]. When excluding from our analysis the first two weeks of the immunisation campaign (during which the maternity teams were adjusting to integrate Nirsevimab in routine patient care), the weekly coverage was higher than 80% and the Nirsevimab effectiveness increased to 84.1%. These results confirm that the risk of RSV‐associated hospitalisation can be minimized with good Nirsevimab implementation. The high immunisation coverage reported in the different studies assessing post‐implementation impact [19, 22, 24], is encouraging of the widespread acceptability of this intervention, the ability to be implemented and its effectiveness.

The Nirsevimab impact we described in this report is limited to the prevention of RSV‐associated hospitalisation. While an effect of the Nirsevimab campaign has also been reflected in the number of ambulatory all‐cause bronchiolitis cases in France [25], the benefit of Nirsevimab needs to be assessed for other RSV‐associated outcomes, such as emergency room and primary healthcare consultations. We expect that the overall impact of Nirsevimab will be greater than that reported in the current literature and our study.

Our study used high‐quality data from RSV‐associated hospitalisations in a hospital where RSV infection was routinely tested for in all infants admitted with respiratory symptoms. Nonetheless, we identified three main limitations. First, infants born at the HCL could have been hospitalised with RSV‐associated LRTI at other facilities, even if the most severe cases were admitted to the HCL because it is the only hospital in the Lyon area with paediatric intensive care unit. However, this limitation applies to each season, not particularly the 2023–2024 season, so the comparison of incident hospitalisations over the time periods should not been affected. Another limitation was that for infants not immunised at birth, immunisation was declared by the parents with the help of the child health record booklet and this could had been underreported. We found no difference in characteristics and therapeutics between immunised and non‐immunised infants, except for age, with hospitalised infants who received Nirsevimab being older. However, infants could have had other risk factors that were not measured in our study.

While the Nirsevimab effectiveness estimated in our study was similar to those achieved in clinical trial settings, our work was based on real‐world evidence using only data collected through routine care.

## Author Contributions

**Cécile Chauvel:** writing – original draft, methodology, visualization, formal analysis. **Côme Horvat:** data curation, investigation, writing – review and editing. **Etienne Javouhey:** writing – review and editing, investigation. **Yves Gillet:** writing – review and editing, investigation. **Juliette Hassenboehler:** investigation, data curation, writing – review and editing. **Claire Nour Abou Chakra:** writing – review and editing, supervision. **Corinne Ragouilliaux:** supervision, writing – review and editing. **Franck Plaisant:** writing – review and editing, supervision. **Dominique Ploin:** data curation, supervision, writing – review and editing, investigation. **Marine Butin:** investigation, writing – review and editing, supervision, data curation. **Jean‐Sebastien Casalegno:** conceptualization, investigation, supervision, writing – review and editing. **Marta Nunes C:** conceptualization, writing – original draft, methodology.

## Ethics Statement

The planning conduct and reporting of studies was in line with the Declaration of Helsinki, as revised in 2013. The study protocol was approved by the institutional review board of the Comité Éthique et Scientifique des Hospices Civils de Lyon on 19/10/2023 (Comité Éthique et Scientifique reference 23_256). In accordance with current French regulations, parents were informed of the retrospective study by postal mail, email or an information note at the time of admission and any refusal expressed by the parents led to exclusion from the study. The database was registered to the French data protection agency according to the MR004‐protocol (CNIL‐ n°23_5256, October 2023).

## Conflicts of Interest

MCN reports grants from Sanofi; and personal fees from Pfizer and Sanofi. EJ reports personal fees from Sanofi. None declared for the other authors.

### Peer Review

The peer review history for this article is available at https://www.webofscience.com/api/gateway/wos/peer‐review/10.1111/irv.70054.

## Supporting information


**Figure S1.** Age distribution of infants in Hospices Civils de Lyon birth cohort at hospitalisation with RSV‐associated lower respiratory tract infections.
**Table S1.** Incidence rate of RSV‐associated lower respiratory tract infection hospitalisation during the first six months of life among the Hospices Civils de Lyon birth cohort for pre‐COVID‐19, 2022–2023 and 2023–2024 seasons.

## Data Availability

The data that support the findings are not publicly available due to potential risks of compromising the privacy of the individual patient.
