# Supplementary material for: Changes in Respiratory Syncytial Virus‐Associated Hospitalisations Epidemiology After Nirsevimab Introduction in Lyon, France
Source: Influenza Other Respir Viruses. 2024 Dec 19;18(12):e70054. doi: 10.1111/irv.70054 (PMC11658964; doi:10.1111/irv.70054)
Supplement: Supplementary file 1 — Figure S1. Age distribution of infants in Hospices Civils de Lyon birth cohort at hospitalisation with RSV‐associated lower respiratory tract infections. Table S1. Incidence rate of RSV‐associated lower respiratory tract infection hospitalisation during the first six months of life among the Hospices Civils de Lyon birth cohort for pre‐COVID‐19, 2022–2023 and 2023–2024 seasons. [file IRV-18-e70054-s001.docx]

Supplementary Figure S1. Age distribution of infants in Hospices Civils de Lyon birth cohort at hospitalisation with RSV-associated lower respiratory tract infections


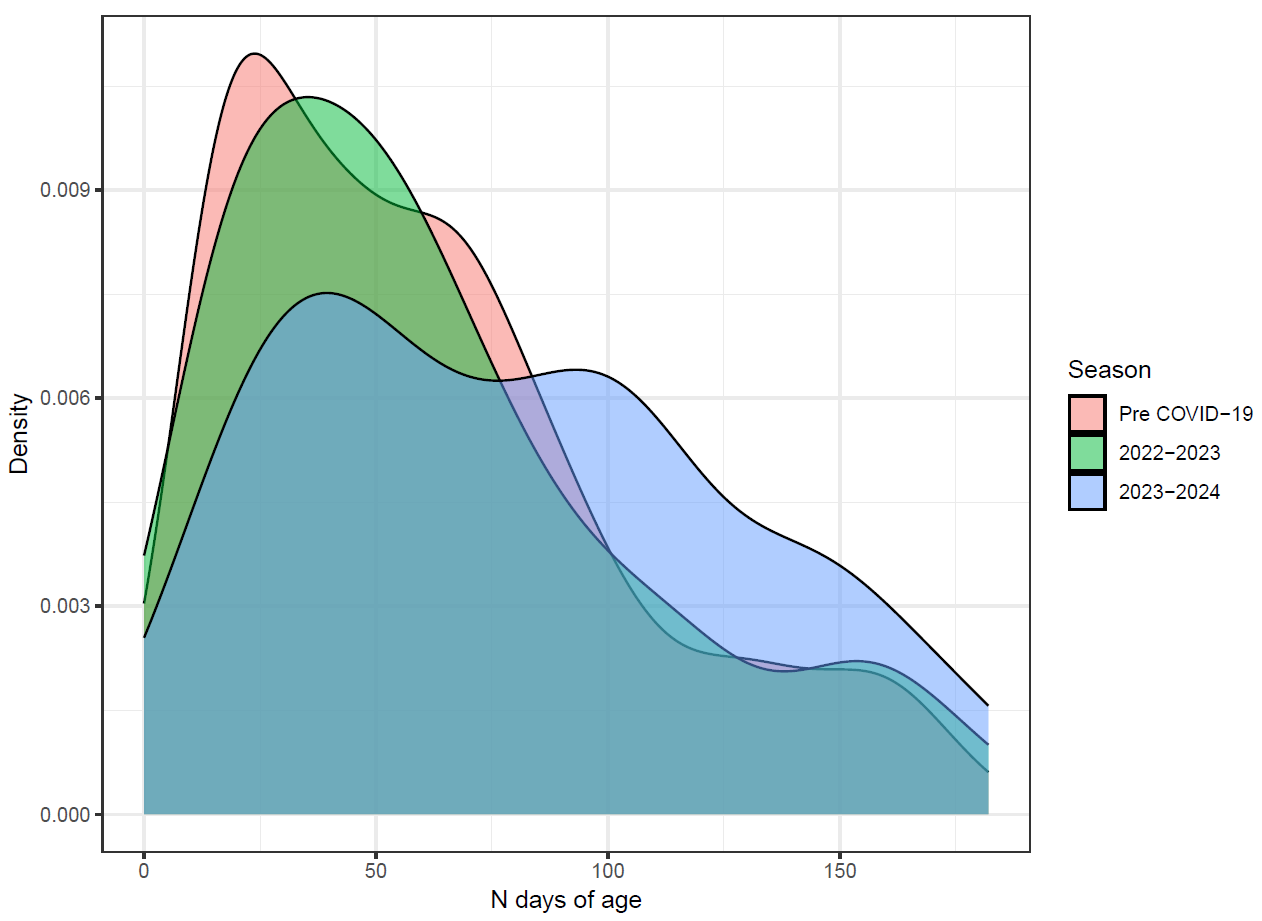


Supplementary Table S1. Incidence rate of RSV-associated lower respiratory tract infection hospitalisation during the first six months of life among the Hospices Civils de Lyon birth cohort for pre-COVID-19, 2022-2023 and 2023-2024 seasons

|  | **Pre COVID-19 seasons** | | **2022-2023**  **season** | | **2023-2024**  **season** | | **Incidence rate ratio** | |
| --- | --- | --- | --- | --- | --- | --- | --- | --- |
| **Delay in months between birth and RSV season onset** | **RSV+ cases** | **Incidence rate per 100 births [95%CI]** | **RSV+ cases** | **Incidence rate per 100 births [95%CI]** | **RSV+**  **cases** | **Incidence rate per 100 births [95%CI]** | **2023-2024 vs**  **pre COVID-19 [95%CI]^§^** | **2023-2024 vs 2022-2023 [95%CI]^§^** |
| **Overall*** | 467 | 3.68 [3.35;4.03] | 83 | 3.12 [2.48;3.86] | 40 | 1.65 [1.18;2.25] | **0.45**  **[0.33;0.62]** | **0.53 [0.36;0.77]** |
| **-2 to -1** | 84 | 3.19 [2.54;3.95] | 12 | 2.02 [1.05;3.53] | 8 | 1.64 [0.71;3.23] | 0.51  [0.25;1.06] | 0.81 [0.33;1.98] |
| **-1 to 0** | 143 | 4.01 [3.38;4.72] | 25 | 3.31 [2.14;4.88] | 15 | 2.26 [1.26;3.72] | **0.56**  **[0.33;0.96]** | 0.68 [0.36;1.29] |
| **0 to 1** | 152 | 4.56 [3.87;5.35] | 26 | 3.96 [2.59;5.80] | 12 | 1.73 [0.89;3.02] | **0.38**  **[0.21;0.68]** | **0.44 [0.22;0.87]** |
| **1 to 2** | 88 | 2.79 [2.24;3.44] | 20 | 3.04 [1.86;4.69] | 5 | 0.88 [0.28;2.04] | **0.31**  **[0.13;0.77]** | **0.29 [0.11;0.77]** |
| **-4 to -3** | 34 | 0.93 [0.64;1.30] | 7 | 0.82 [0.33;1.69] | 12 | 1.84 [0.95;3.21] | **1.98**  **[1.02;3.82]** | 2.25 [0.89;5.71] |
| **-3 to -2** | 55 | 1.56 [1.18;2.03] | 6 | 0.69 [0.25;1.51] | 16 | 2.61 [1.49;4.23] | 1.67  [0.96;2.91] | **3.77 [1.47;9.62]** |

*For 2023-2024 season, overall includes all infants born from September 18 to December 31, 2023 (-2 to 2 months).

**^§^**In bold, significant results.
